# Infrared stimulated emission with an ultralow threshold from low-dislocation-density InN films grown on a vicinal GaN substrate

**DOI:** 10.1016/j.fmre.2021.09.020

**Published:** 2021-12-18

**Authors:** Huapeng Liu, Bowen Sheng, Tao Wang, Konstantin Kudryavtsev, Artem Yablonskiy, Jiaqi Wei, Ali Imran, Zhaoying Chen, Ping Wang, Xiantong Zheng, Renchun Tao, Xuelin Yang, Fujun Xu, Weikun Ge, Bo Shen, Boris Andreev, Xinqiang Wang

**Affiliations:** a State Key Laboratory for Mesoscopic Physics and Frontiers Science Center for Nano-optoelectronics, School of Physics, Peking University, Beijing 100871, China; b Institute for Physics of Microstructures, Russian Academy of Sciences, Nizhny Novgorod 603950, Russia; c Collaborative Innovation Center of Quantum Matter, Beijing 100871, China

**Keywords:** InN, Stimulated emission, Vicinal substrate, Phonon replica, Molecular beam epitaxy

## Abstract

Near-infrared stimulated emission from a high-quality InN layer under optical pumping was observed with a threshold excitation power density of 0.3 and 4 kW cm^−2^ at T = 8 and 77 K, respectively. To achieve such a low threshold power density, vicinal GaN substrates were used to reduce the edge-component threading dislocation (ETD) density of the InN film. Cross-sectional transmission electron microscopy images reveal that the annihilation of ETDs can be divided into two steps, and the ETD density can be reduced to approximately 5 × 10^8^ cm^−2^ near the surface of the 5-µm-thick film. The well-resolved phonon replica of the band-to-band emission in the photoluminescence spectra at 9 K confirm the high quality of the InN film. As a result, the feasibility of InN-based photonic structures and the underlying physics of their growth and emission properties are demonstrated.

## **Graphical abstract**

**Figure:**
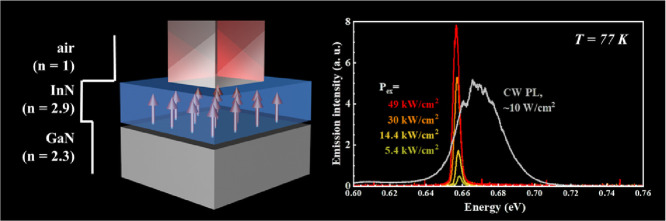

## 1 Introduction

Luminescence features (optical properties) are known to be strongly dependent on the crystal quality. For instance, the bandgap of InN, which was previously accepted as approximately 2 eV [1,2], has been revised to 0.64 eV [3], [4], [5], [6], [7], [8], [9] after a breakthrough in InN epitaxial technology. The newly determined bandgap makes InN a promising candidate for developing optoelectronic devices, such as infrared (IR) sensors, light-emitting diodes, and lasers, and introduces further prospects for III-nitrides in next-generation communication techniques [10], [11], [12]. However, poor quality InN layers, not only prevent the realization of these devices with their anticipated performance, but hinder a thorough understanding of their fundamental structure and optical properties. For example, phonon replica peaks have previously been extensively observed in wurtzite crystal structures [13,14]; however, they are difficult to observe in InN films because of the difficulty in obtaining high-quality InN. A reason for this is the high free-carrier concentration in InN, which seriously affects the photoluminescence (PL) energy and line-width through band filling [15], making it difficult to observe phonon replica peaks [5]. Recently, near-infrared stimulated emission from InN and In-rich InGaN layers was observed, demonstrating the feasibility of InGaN-based lasers as well as the potential of crystalline indium nitride as a photonic material [12,16]. Basic conditions for the realization of stimulated emission in planar InN structures include the following: low electron concentration, low dislocation density, homogeneity of the InN layer, absence of the metallic In phase, and the low density of the absorption and nonradiative recombination centers. To attain stimulated emission, a high-quality InN film is a prerequisite. It has been reported that the use of a vicinal substrate is an effective technique for reducing the threading dislocation (TD) density in GaN and AlN [17], [18], [19], [20], [21], [22]. However, this technique has not yet been applied to the growth of InN films.

In this study, high-quality InN films with a low dislocation density of 5 × 10^9^ cm^−2^ and a low electron concentration of 3.6 × 10^17^ cm^−3^ were produced using a vicinal GaN substrate. Cross-sectional transmission electron microscopy (TEM) images show that the reduction mechanism of the edge-component TD (ETD) density is divided into two steps, and the ETD density can be eventually annihilated to approximately 5 × 10^8^ cm^−2^. Temperature-dependent PL measurements were also performed with a clear sideband emission peak (SBEP) observable up to 150 K, which were identified as phonon replica of the band-to-band emission peak. Relying on low-dislocation density and low electron concentration, stimulated emissions with an ultralow threshold pump power density at direct band-to-band transitions in InN were observed under optical excitation. The superior emission properties of InN films grown on vicinal GaN substrates signify the potential of InN materials for infrared solid-state lasers.

## 2 Materials and methods

### 2.1 Sample preparation and characterization

In-polar InN films were grown by plasma-assisted molecular beam epitaxy (SVTA) on GaN (0001) substrates with vicinal angles varying from 0.35° to 2.0° towards the m-direction. The growth temperature was controlled just below the boundary temperature for dissociation to improve the crystal quality [23], and the In/N ratios were maintained under a slightly In-rich condition [23,24]. The surface and quality of the samples were analyzed by atomic force microscopy (AFM) and high-resolution X-ray diffraction (HRXRD), respectively. Furthermore, the electron concentration of the samples was determined using room-temperature Hall-effect measurements, and cross-sectional TEM was used to characterize TD behaviors. Temperature-dependent PL measurements were performed using a semiconductor diode laser with a wavelength of 532 nm and power of 80 mW for excitation. The samples were mounted in a continuous-flow liquid-helium Dewar, and a Fourier transform infrared (FTIR) spectrometer-based PL system was set in the continuous-scan mode. PL emission was detected using a cooled InSb photodetector.

### 2.2 Emission measurements

An optical parametric oscillator (Spectra Physics MOPO-SL) was chosen as the excitation source, which provided pulses lasting 10 ns at a repetition frequency of 10 Hz. The sample's emission was dispersed using a 300-mm grating spectrometer (Princeton Instruments Acton-2300i) and detected using an LN-cooled extended range InGaAs diode array (OMA-V). Measurements were performed with samples placed inside the LN Dewar vessel; the excitation beam covered the entire sample surface. The emitted light was collected from the surface (not from the edge) of the samples.

## 3 Results and discussion

**Fig. 1:**
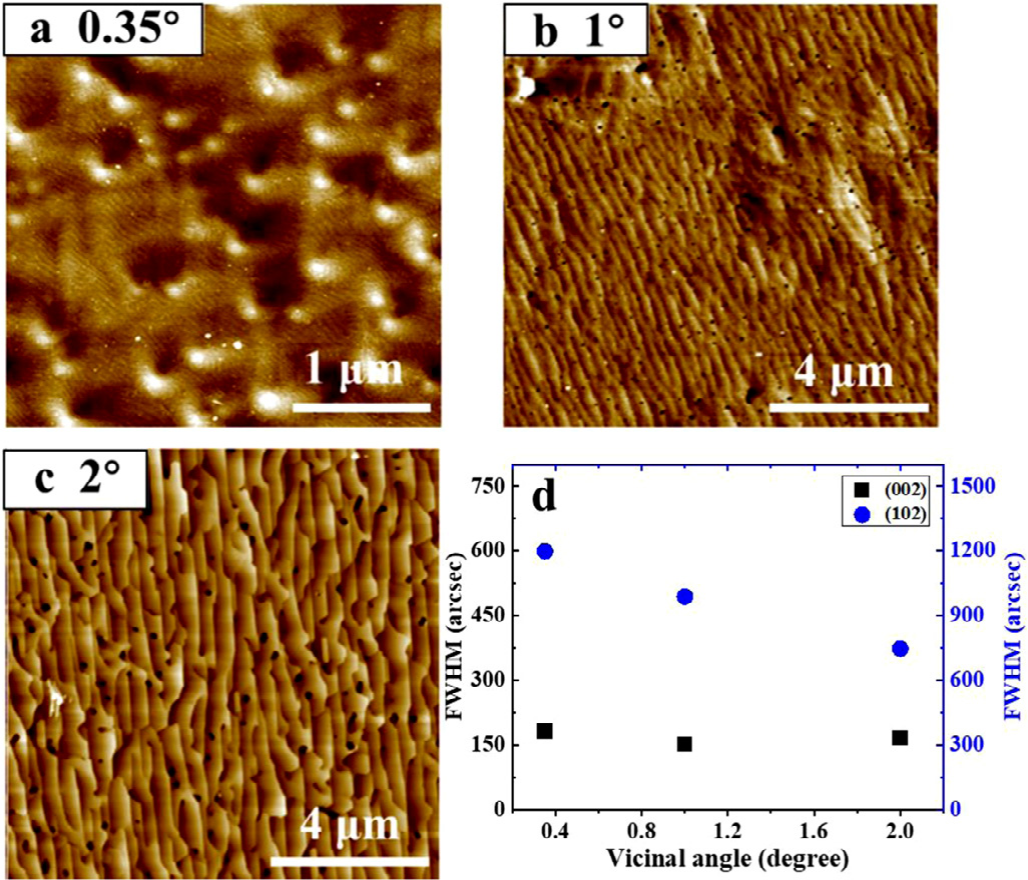
**AFM surface images of InN grown on vicinal GaN substrates**. (a) 0.35°-off, (b) 1°-off and (c) 2°-off. (d) The full width at half maximum (FWHM) of the X-ray diffraction rocking curves measured for the (002) and (102) diffraction peaks of InN films grown on GaN substrates with different vicinal angles.

InN surface morphologies are shown in Fig. 1a–c, which clearly indicate a strong dependence on the vicinal angle of the substrate. When the vicinal angle was 0.35°, monolayer steps with hexagonal spiral features were observed, as shown in Fig. 1a; the surface was ultra-flat with a root mean square (rms) roughness of 0.38 nm in a 3 μm × 3 μm scanned area. On the other hand, when the InN films were grown on substrates with larger vicinal angles, well-ordered straight macro-step features were clearly observed without spiral features, as shown in Fig. 1b, c. The widths of the macro-steps are different in Fig. 1b, c. When InN was grown on the 1°-off vicinal substrate, the macro-steps were approximately 1 nm in height and 200 nm in width, whereas when the InN was grown on the 2°-off vicinal substrate, much larger macro-steps were observed with a height and width of 5.4 nm and 350 nm, respectively. The formation of macro-steps implies that the reduction in TD density occurred, as reported in previous studies [20,21,25] which is supported by the X-ray diffraction (XRD) measurements shown in Fig. 1d. The full width at half maximum (FWHM) values for the (102) diffraction peaks decreased from 1200 to 750 arcsec as the vicinal angle increased from 0.35° to 2°, which indicates an improvement in the twisting feature of the InN film grain structure. However, the FWHM values for the (002) diffraction peaks were almost the same within the range of 170 ± 10 arcsec, which is much smaller than those grown on sapphire and GaN/sapphire templates [26,27]. The small FWHM value for the (002) diffraction peak demonstrates that the screw-component threading dislocations (STDs) are mainly dependent on the STDs of the GaN substrate. It is well known that the FWHM of the XRD rocking curves on the InN (002) and (102) planes can be used to estimate the dislocation density through the mosaic model [28]. From this, the STD and ETD densities were estimated to be approximately 4.4 × 10^7^ cm^−2^ and 5 × 10^9^ cm^−2^, respectively.

**Fig. 2:**
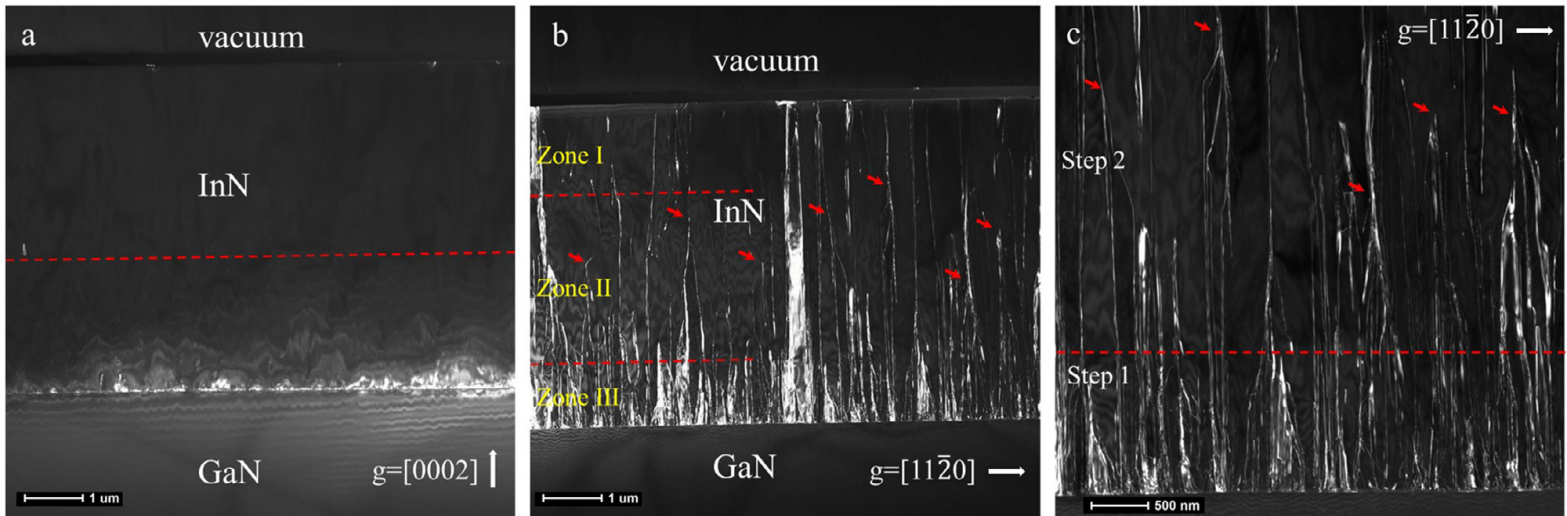
**Cross-sectional dark-field transmission electron microscopy (TEM) images of the InN epilayer grown on a 2.0°-off vicinal substrate.** (a) g = [0002] and (b) [11$\bar{2}$0]. (c) Magnified cross-sectional dark-field TEM, g = [11$\bar{2}$0].

A cross-sectional dark-field TEM experiment was performed to understand the TD behavior in more detail. In Fig. 2a, no STDs are observed along the dashed line, indicating that the STD density is less than 1 × 10^8^ cm^−2^; this is consistent with the XRD results. On the other hand, as shown in Fig. 2b, the ETDs have a non-uniform distribution along the growth direction. The ETDs were classified into three zones and annihilated in two steps. In the bottom zone (within a thickness of 1 μm), the ETD density was very high because of the large lattice mismatch between the InN film and the GaN buffer layer/template (∼11 %). The ETDs were annihilated primarily in this zone owing to the obstruction of the vertical TDs when they encountered the inclined TDs, as revealed by the magnified TEM image (Fig. 2c). This annihilation mechanism was also reported by Shen et al. and the reduction process is referred to as step 1 [20,21]. After step 1, there was a possibility for several of the remaining ETDs to combine with each other to further reduce the ETD density. The clustered ETDs are indicated by red arrows in Fig. 2c. This process, known as step 2, occurred at a thickness of 1.5–3.5 μm (zone II), and the ETD density in zone II was estimated to be 2.5 × 10^9^ cm^−2^. Finally, the ETDs rose to the surface with an average spacing that was larger than the width of the macro-steps, and the ETD density in this top zone was estimated to be 5 × 10^8^ cm^−2^.

**Fig. 3:**
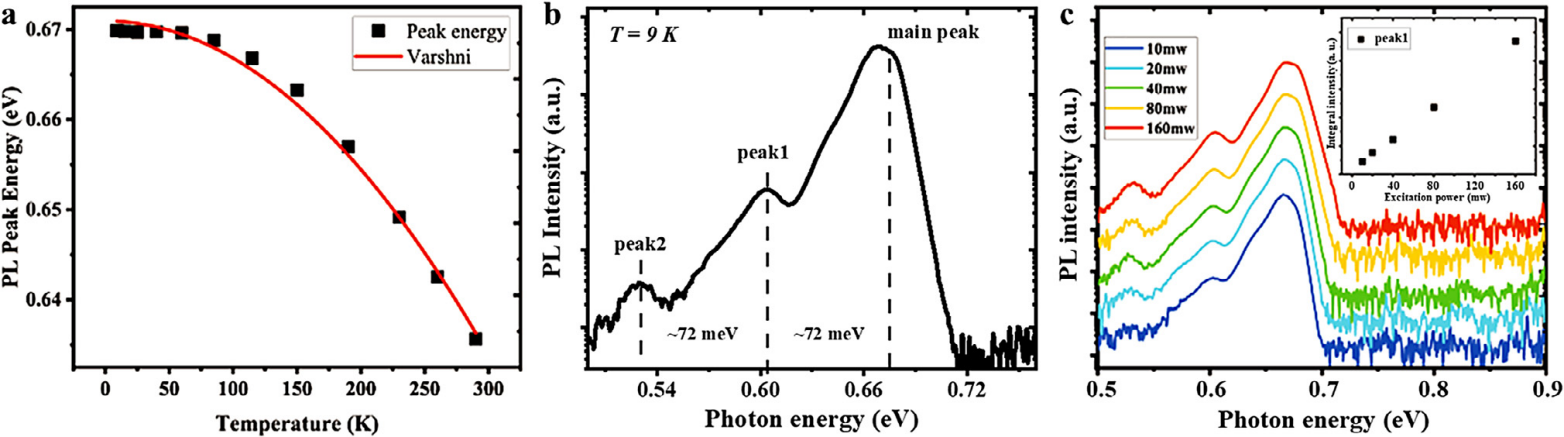
**Photoluminescence (PL) of InN film.** (a) Variation of the PL peak energy with temperature and the fit using the Varshni equation (solid line). (b) Low temperature (9 K) PL spectra of InN film grown on 2°-off GaN substrate. (c) PL spectra of InN film under different excitation powers at 9 K. The inset is the dependence of the integrated intensity of peak1 on excitation power.

Subsequently, temperature-dependent InN PL spectra with the lowest dislocation density were measured from 9 K to room temperature, and a well-resolved emission consisting of three peaks was observed. In Fig. 3a, the dominant PL peak shifts from ∼0.669 eV (at 9 K) to ∼0.635 eV (at room temperature). The clear redshift of the main PL peak energy with increasing temperature can be effectively described by the Varshni equation [6] *:*

(1) $$E_{g}\left(T\right)=E_{g}\left(0\right)--\alpha T^{2}/\left(\beta +T\right)$$

where *E_g_*(0) corresponds to the PL peak energy at *T* = 0 K, coefficient *α* is related to the thermal expansion, and *β* is loosely associated with the Debye temperature [5]. The fitting parameters, *E_g_*(0) = 0.672 ± 0.001 eV, *α* = 0.376 ± 0.02 meV K^−1^, and *β* = 597 ± 38 K, are in agreement with published values obtained from high quality InN films [5,6]. According to previous studies (for example, Klochikhin et al. [29]), the main PL peak is a complex band that combines contributions from pure band-to-band recombination and electron transitions of from the bottom of the conduction band to both the shallow acceptor states with a binding energy of 9 meV and the Urbach tail states. On the low-energy side of the PL spectra, two weaker PL peaks appear at approximately 0.603 eV and 0.532 eV (at 9 K) with an energy separation of ∼72 meV from the band-to-band emission peak. This separation is consistent with the theoretically predicted longitudinal optical phonon energy [30]. To identify whether these bands are phonon sidebands (PSBs), the regular spacing and their temperature dependence are investigated, as described below. In addition, as shown in Fig. 3c, all three peaks become stronger with increasing pumping power, and the excitation intensity of the low-energy feature at 0.603 eV does not saturate with increasing excitation power. Therefore, this rules out the possibility that the low-energy PL emission is due to a deep acceptor energy level transition, as reported by Klochikhin et al [29].

**Fig. 4:**
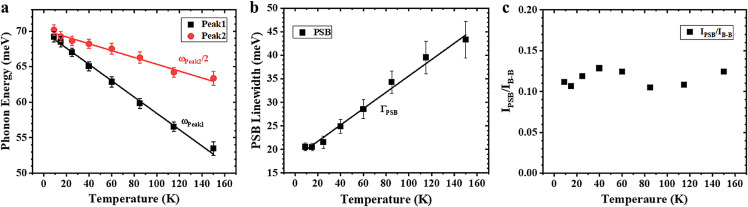
**Temperature dependent phonon sideband (PSB) properties.** (a) The linear fits of temperature dependent phonon energy. (b) The linear fit of temperature dependent PSB linewidth. (c) The ratios of emission intensity between phonon replicas and the band-to-band emission peaks.

To verify whether the PSBs are phonon replicas of the band-to-band emission peak, the temperature-dependent energy and linewidth of the weaker PL peaks were investigated, and the energy and linewidth of these peaks presented linear relationships with temperature (9–150 K). Temperature-dependent PSB properties have been experimentally studied for GaN [13] and InN epilayers [5], where the energy shift with temperature is expressed as [5,31]:

(2) $$\omega_{\text{PSBm}}\left(T\right)=m\omega_{0}\;\text{--}\;\left(\frac{5}{2}\;\text{--}m\right)k_{B}T\;\text{--}m\Delta \left(T\right).$$

where *m* is the PSB index (*m* = 1, 2), *ω*_0_ is the PSB energy at *T* = 0 K, and the third term results from the anharmonic shift due to phonon decay, which is negligible compared with the linear term [5,13]. From this, the theoretical values for *ω*_PSB1_ and *ω*_PSB2_ were calculated as -0.129 and -0.043 meV K^−1^, respectively. The linear fits of the temperature-dependent PSB energy, as shown in Fig. 4a, produced slopes of (-0.115 ± 0.003) and (-0.095 ± 0.005) meV/K for *ω*_peak1_ and *ω*_peak2_, respectively. It is clear that only peak1 is consistent with the theoretical predictions. PSB linewidth (Γ_PSB_) also evolves with temperature, as expressed by [5,31]:

(3) $$\Gamma_{\text{PSBm}}\left(T\right)=\;2\sqrt{\left(\frac{5}{2}\;-\;m\right)}k_{B}T+\Gamma \;\left(T\right)+\Gamma_{\text{phonon}}\;\left(T\right).$$

where Γ(*T*) is the temperature-dependent broadening of the interband emission peak caused by the electron-phonon interaction, and Γ_phonon_(*T*) is the temperature-dependent phonon broadening, which is considered negligible in this case [5,13]. The plot of the PSB linewidth against temperature is shown in Fig. 4b. The linear fits of Γ_PSB_(*T*) produced slopes of (0.194 ± 0.009) meV K^−1^ for Γ_peak1_, which is consistent with the calculated temperature coefficients (0.211 meV K^−1^ for Γ_PSB1_). Furthermore, PSBs are distinguished by the Huang-Rhys factor, which can be calculated by the ratios of emission intensity between phonon replicas and the band-to-band emission peaks [32]. As shown in Fig. 4c, the calculated Huang-Rhys factors are almost constant, which is consistent with Huang-Rhys theory. Therefore, peak1 can be identified as phonon replica of the band-to-band emission peak.

**Fig. 5:**
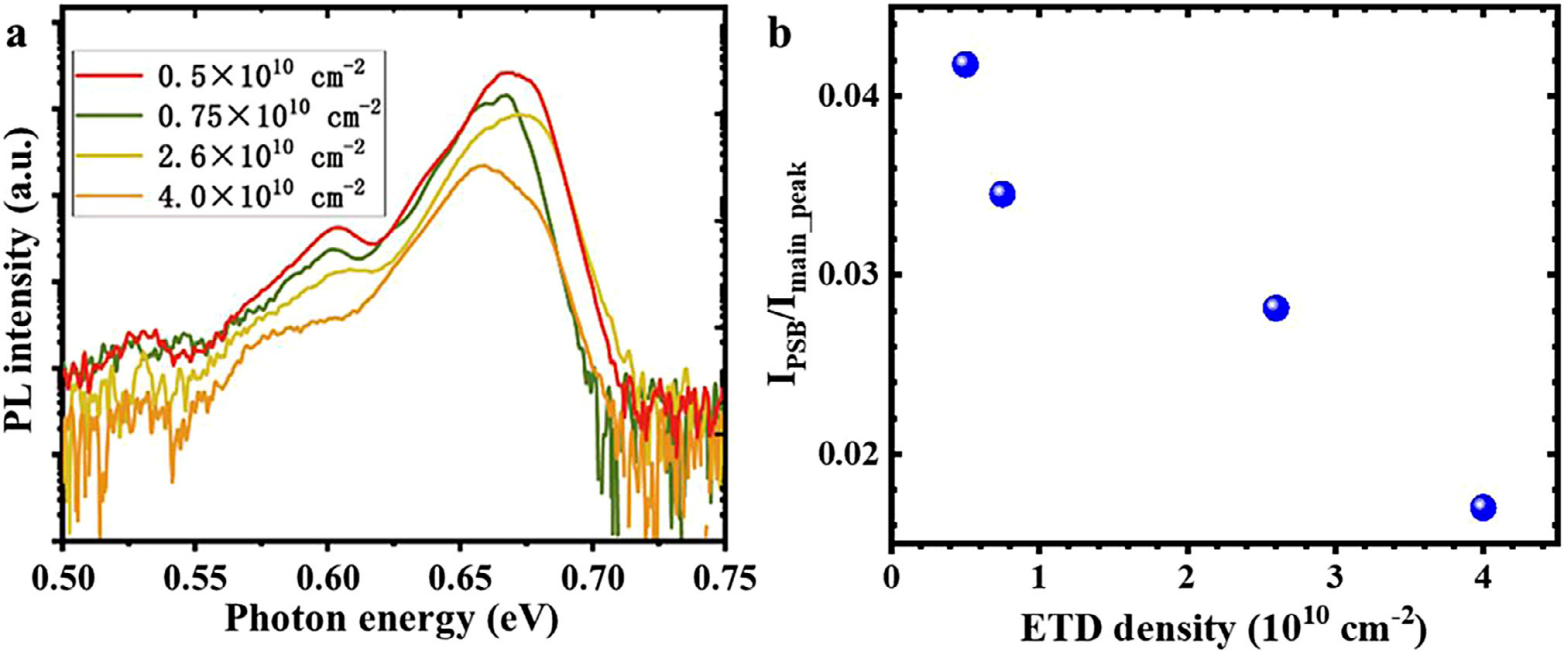
**The phonon sideband emission peaks related to the edge-component threading dislocation (ETD) density.** (a) The photoluminescence characteristics of InN with different ETD densities. (b) The dependence of the I_PSB_/I_main_peak_ ratio on the ETD densities.

Finally, the appearance of the PSB emission peak was found to be related to the low ETD density, and Fig. 5a clearly shows that the sample with the lowest ETD density has the most distinct PSB emission peaks. To illustrate their relationship in detail, the ratio of integrated intensity between the PSB peak and the main PL peak (I_PSB_/I_main___peak_) was calculated for different ETD densities. In Fig. 5b, the I_PSB_/I_main_peak_ ratio decreases with increasing ETD density, which indicates that ETDs in InN may behave as nonradiative recombination centers, capturing carriers from recombination [33]. Therefore, the PSB emission intensity is strongly associated with the ETD density.

**Fig. 6:**
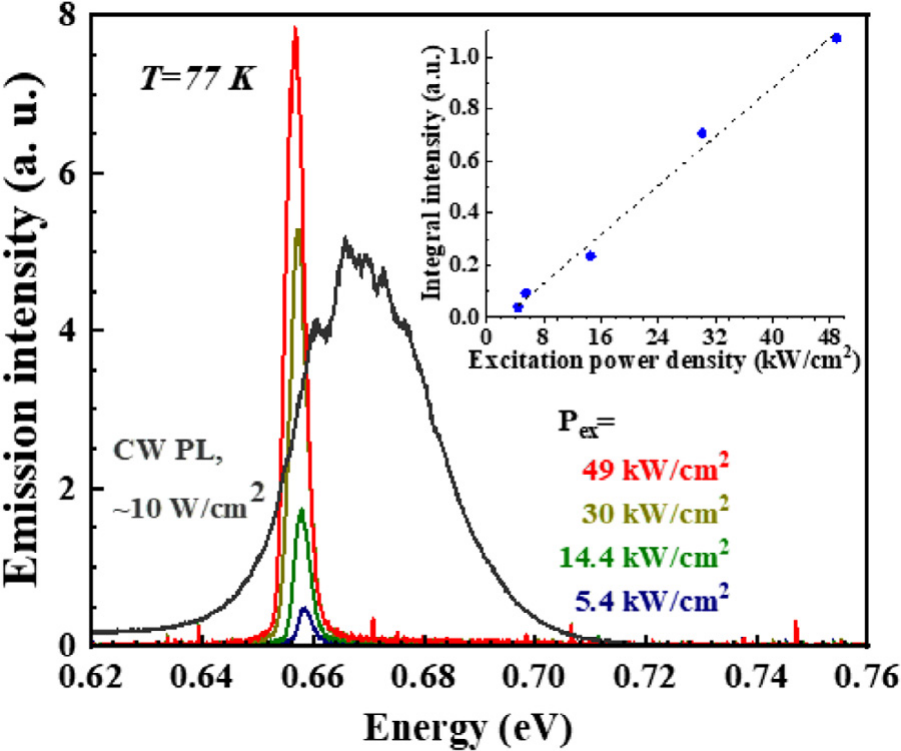
**Emission spectra measured at different excitation power densities (color lines,**λ**_ex_ = 680 nm).** Spontaneous emission spectrum measured under CW excitation is shown for reference (gray line). All measurements were taken at T = 77 K. Inset: dependence of integral intensity on the pump power density.

The emission spectra were studied under optical excitation using a continuous wave (CW) laser and an optical parametric oscillator, tunable in the spectral range 0.45 to 2.3 μm (up to 3 mJ in 10 ns pulses with a repetition rate of 10 Hz). The emitted radiation was collected from the surface or from the edge of the sample. In the case of pulsed excitation, the pump wavelength was tuned to achieve uniform excitation across the entire active layer for samples with thick InN layers. Further details can be found in the Methods section and other sources [12]. Fig. 6 shows the sample's PL spectra at the temperature of liquid nitrogen (T = 77 K). The obtained sample demonstrated broad, intense, spontaneous PL (gray line) from the InN layers under CW optical pumping.

Under pulsed excitation, a relatively narrow line arose at approximately 1.88 µm for pump power densities exceeding the threshold (∼4 kW cm^−2^ at T = 77 K). The emission linewidth just above the threshold was approximately 7 nm (2.5 meV), compared with ∼100 nm (∼35 meV) for spontaneous PL. The intensity of this line demonstrates a superlinear dependence on the pump power (inset in Fig. 6), which clearly indicates stimulated emission at the interband transitions in degenerate InN with an electron concentration of ∼10^17^ cm^−3^. At T = 8 K, the threshold pump power density for the sample was estimated to be P_th_ = 300 W cm^−2^. The threshold of stimulated emission in the obtained planar structures was lower than the previously reported value for samples with an InN layer [12].

## 4 Conclusion

In summary, stimulated emission from a low-dislocation density InN was obtained under optical excitation. The stimulated emission threshold was 0.3 kW cm^−2^ and 4 kW cm^−2^ at T = 8 and 77 K, respectively. The high-quality InN film was grown on a 2°-off vicinal GaN substrate with an electron concentration of 3.6 × 10^17^ cm^−3^ and ETD density of 5 × 10^9^ cm^−2^. Cross-sectional TEM images show that the reduction of ETDs can be divided into two steps, and the ETD density can be annihilated to approximately 5 × 10^8^ cm^−2^. The temperature-dependent PL spectra of the low-ETD-density InN film show a dominant PL emission peak containing three peaks and clear discrete SBEP. The SBEP emission is identified as phonon replica by the Huang-Rhys factor and prior theory [34] of temperature-dependent E_phonon_ and Γ_PSB_. Moreover, it was found that the ETD density should be low to detect PSB emissions.

## Declaration of Competing Interest

The authors declare that they have no conflicts of interest in this work.
